# Protist diversity and community structure around the Xianbei Seamount in the South China Sea

**DOI:** 10.1128/spectrum.02734-25

**Published:** 2025-11-24

**Authors:** Wenxue Wu, Sicheng Yao, Xinke Li, Hailong Huang, Ting Yan, Xinghai Yang, Lei Wang, Wei Xie, Haibo Jiang

**Affiliations:** 1 State Key Laboratory of Marine Resource Utilization in South China Sea, School of Marine Sciences, Hainan University 74629, Haikou, China; 2 Southern Marine Science and Engineering Guangdong Laboratory (Zhuhai) 590852 , Zhuhai, China; 3 School of Ecology, Hainan University 74629, Haikou, China; 4 School of Marine Sciences, Ningbo University 631318 , Ningbo, China; 5 Key Laboratory of Marine Ecological Conservation and Restoration, Third Institute of Oceanography, Ministry of Natural Resources P.R.C. 118477 , Xiamen, China; 6 School of Marine Sciences, Sun Yat-sen University 626303 , Zhuhai, China; Connecticut Agricultural Experiment Station, New Haven, Connecticut, USA

**Keywords:** protist, diplonemid, metabarcoding, quantitative polymerase chain reaction, seamount

## Abstract

Seamounts are topographic rises of the seabed that represent one of the most common underwater ecosystems worldwide. Marine protists comprise various species with complex ecological functions. However, protist communities in seamount-associated ecosystems have not been adequately studied. In this study, we investigated planktonic protist communities around the Xianbei Seamount in the South China Sea. The entire protist community was profiled using universal primers for the 18S rRNA gene, whereas diplonemids (i.e., partial protist communities) were specifically explored using group-specific primers, and the cell numbers of the diplonemids were quantified based on copy numbers. We found that the alpha diversity (richness and Shannon indices) of the entire and partial protist communities significantly decreased and increased, respectively, across the water columns. The water depth-regulated patterns of beta diversity were remarkable, and a depth–decay relationship of community similarity was observed for both categories. Moreover, all four families of diplonemids (Eupelagonemidae, Hemistasiidae, DSPD II, and Diplonemidae) were recovered from water masses around the seamount, and the diplonemid assemblages showed horizontal and vertical changes in cell numbers (ranging from 1.8 × 10^3^ to 5.4 × 10^4^ cells/L). In summary, this study sheds light on the diversity and compositional structures of both entire and partial (i.e., diplonemid-related) protist communities in pelagic ecosystems, with an emphasis on seamount-associated areas.

**IMPORTANCE:**

This study provides a reference for protist communities around the Xianbei Seamount in the South China Sea. Protists are characterized by their vast diversity and fundamentally distinct functions, whereas studies on subclades performed to obtain a better understanding remain scarce. In particular, we explored the diversity and distribution of diplonemids, which have previously been characterized as the most species-rich lineage. Our results, based on environmental DNA metabarcoding using the newly designed primers, support the great diversity of diplonemids. We further quantified the cell number of diplonemids based on the copy number of the 18S rRNA gene and determined the horizontal and vertical variations around the seamount. In summary, in this study, we not only describe protist communities in rarely investigated habitats (i.e., seamount-associated ecosystems) but also provide new information on diplonemids that apparently act as the dark taxa of marine protists.

## INTRODUCTION

Seamounts are topographic rises on the seafloor and represent one of the most common underwater ecosystems on Earth (1). Seamounts were originally defined as having an elevation of more than 1,000 m above the seabed; however, the term seamount is also used to describe geomorphological features higher than 100 m in elevation (2). The area of global seamounts reaches 28.8 million km^2^ (based on a total of 44,800 seamounts) (3), wherein spatial and environmental heterogeneities arise at the seascape level (4). Therefore, the seamount biome is a biodiversity hotspot (5) with high variability, as revealed by large-scale censuses (6). Considering marine life present over seamounts, studies on megabenthos have mainly evaluated the effects of human activities, such as seabed mining and bottom trawling (7). For nektonic organisms, a few mechanisms, such as the stepping-stone hypothesis (8), have been specifically established to interpret spatial patterns among seamounts (9). Planktonic communities can be disproportionately productive above seamounts, which pump nutrient-rich deep water into the upper layers (10), causing the seamount effect (7). Remarkably, seamounts have been found to affect both phytoplankton (11) and zooplankton (12).

Protists are extremely diverse across soil, freshwater, and marine environments (13) and are functionally important in seamount ecosystems. For example, xenophyophores (Foraminifera, Rhizaria), which are giant sediment-agglutinating protists, are dominant epifauna on many bathyal seamounts (14). They provide habitats for numerous metazoan taxa on seamounts (15), a function that can be extended to fish nurseries (16). The role of xenophyophores in maintaining abyssal biodiversity has been well documented, despite poor knowledge of their biology (17). Ciliates can be unusually diverse, accompanied by apparent patchiness, and can increase the planktonic standing stock in relation to the productivity around seamounts (18). This observation is supported by a recent study showing that, compared with physicochemical conditions, ciliates provide higher-resolution indications of the seamount effect (19). Thraustochytrids (Labyrinthulomycetes, Stramenopila) isolated from the hydrothermal vents of the D. João de Castro Seamount showed growth optimization linked to a few trace metals (20), suggesting that protist communities can be intricately tied to seamount characteristics (e.g., elemental stoichiometry). Although relevant pilot studies have been conducted, most protists found in seamount ecosystems have not been sufficiently studied.

Diplonemids (Diplonemea, Excavata) are dark taxa (21) with high diversity and a limited number of described species (22). Since being first documented more than a century ago, this lineage has been considered a minor fraction of planktonic protists (23). Correspondingly, diplonemids had been sporadically recorded in some pioneering studies exploring marine protists through 18S rRNA gene sequencing (24), followed by the establishment of a phylogenetic context (25). Among the clones detected in oceans globally, diplonemids have a relative abundance of less than 1.4% (26, 27). The *Tara* Oceans expedition considerably altered our understanding of the role of diplonemids by revealing their remarkably high richness (28), which has rendered them apparently dark taxa (21), as they are the most species-rich clade among protists (29). Subsequently, the morphological identification of several diplonemid representatives helped strengthen their diversity, which was previously restricted to environmental DNA (eDNA) metabarcoding of a single gene (i.e., 18S rRNA gene). Moreover, their heterotrophic modes have been verified using single-cell genomics (30) and transcriptomics (31). Until recently, a few advances have been made regarding the ecological characteristics of diplonemids in terms of their ultrastructure, life cycle, feeding strategy, metabolism, and endosymbionts (32). With many special characteristics, diplonemids can be used as indicator taxa in deep waters (33), and their indicative role may be enhanced in habitats with strong vertical gradients (e.g., around seamounts). However, in contrast to taxonomic diversity, compositional variation within diplonemids has rarely been examined, although regionally divergent patterns have been observed (34, 35).

**Fig 1:**
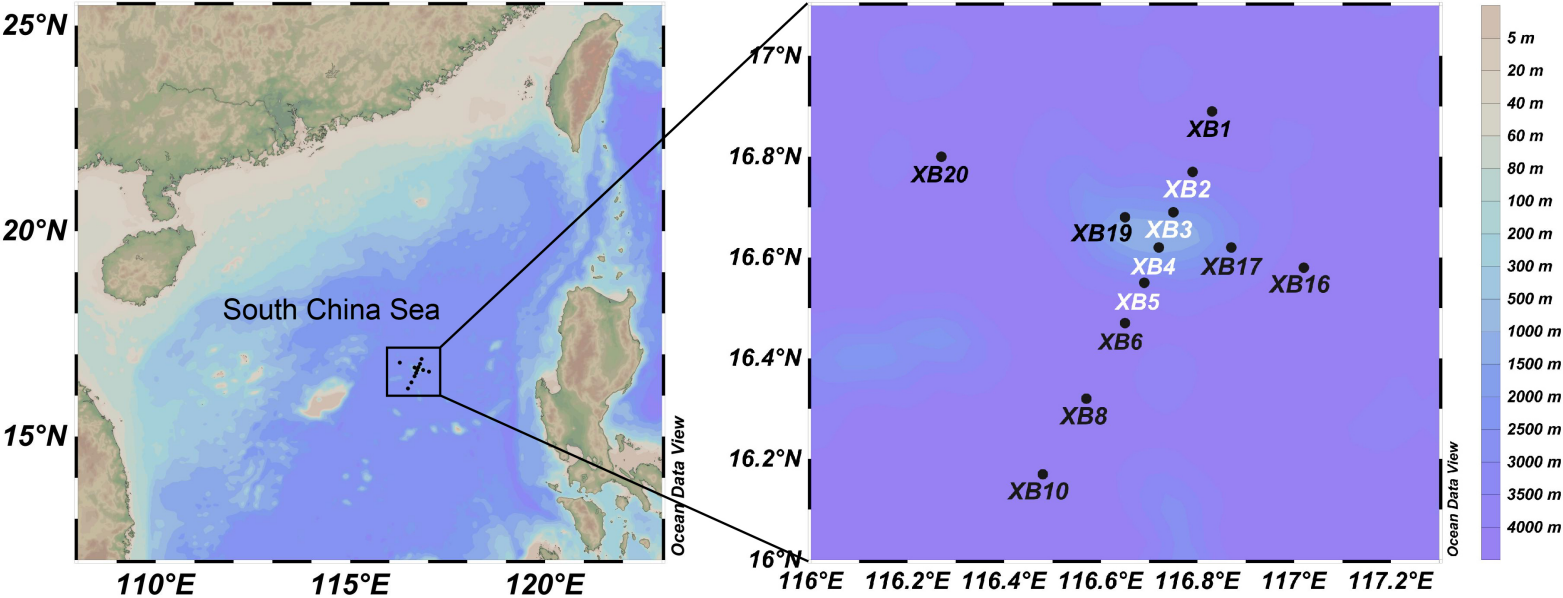
Geographic location of sampling stations (indicated by solid dots) around the Xianbei Seamount. The four stations (XB2–XB5) surveyed vertically are marked with names presented in white. This map was generated using the Ocean Data View software (http://odv.awi.de).

To date, very few seamounts have been studied using integrative approaches (36–38), and a knowledge gap exists in protist studies, partly because protistologists rarely have the opportunity to investigate protist communities over seamounts. In this study, we surveyed the Xianbei Seamount in the South China Sea (Fig. 1) as part of a multidisciplinary expedition (39, 40). The Xianbei Seamount is a relatively shallow seamount with a summit depth of 208 m and a height of approximately 3,800 m. Prior analyses confirmed a vertically promoted transport loop for organic carbon, indicating a seamount effect (40). We used universal (41) and diplonemid-specific 18S rRNA gene primers (42) to characterize the entire and partial planktonic protist communities, respectively. In particular, we evaluated the effect of water depth on alpha- and beta-diversity-related patterns around the seamount. We then explored the phylogenetic structures of diplonemids derived from the group-specific primer set. Moreover, we explored the copy numbers of diplonemids using quantitative polymerase chain reaction (qPCR) to obtain copy number-revised community structures and cell numbers. Overall, our study provides a baseline for protist diversity and community structure in seamount ecosystems, where knowledge of conservation and management is urgently required (43).

## RESULTS

### Community characterization

**Fig 2:**
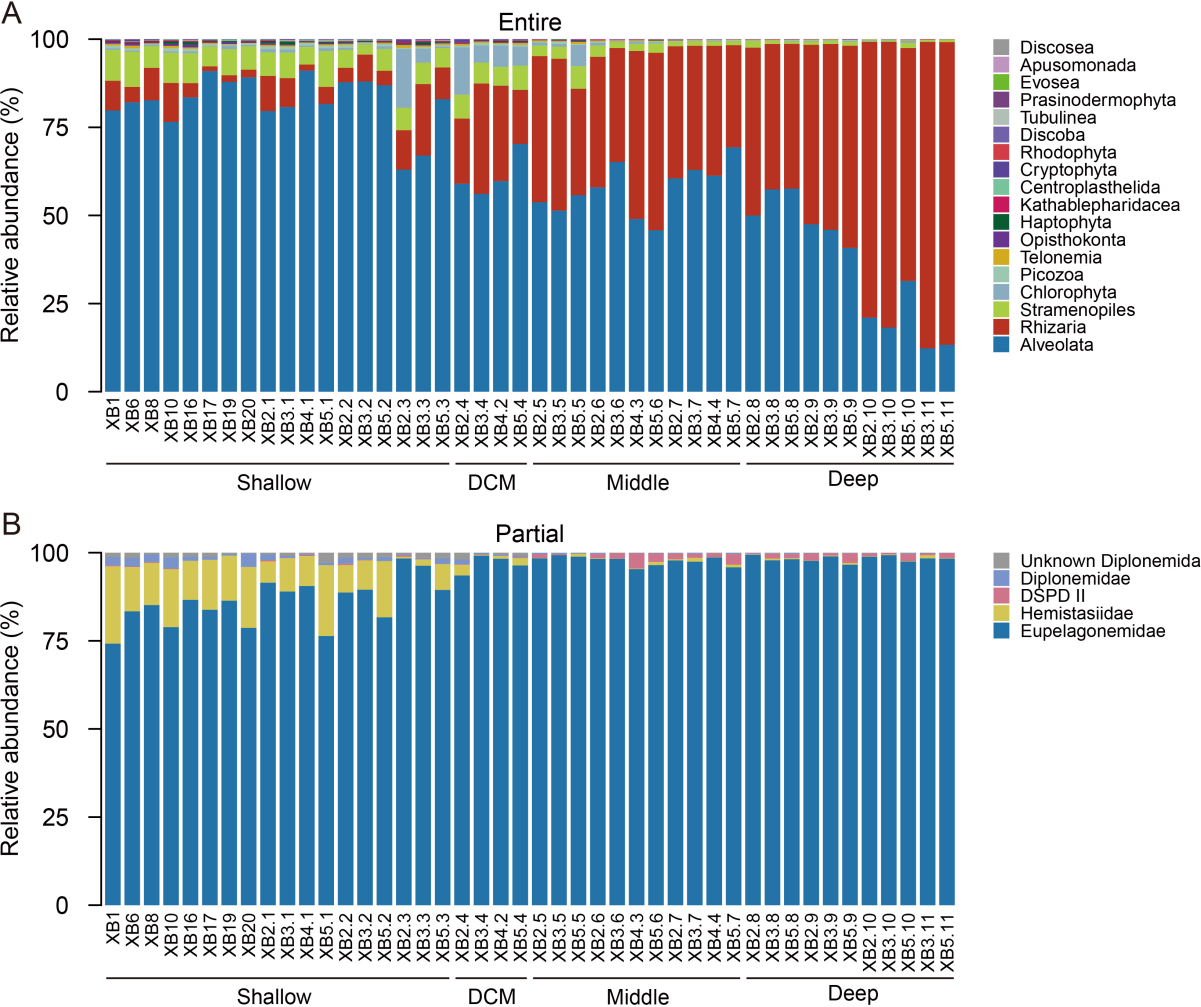
Compositions of entire (**A**) and partial (**B**) protist communities, presented as the relative abundance (%) of taxa at the third rank (in the PR^2^ database) and family level, respectively.

In total, 2,973,145 quality-filtered sequences were retained for protist communities derived from universal primers, generating 28,694 amplicon sequence variants (ASVs) (Table S1). The SAR clade (i.e., Stramenopiles, Alveolata, and Rhizaria) was dominant, and the three subclades showed average relative abundances of 4.1% (Stramenopiles), 62.6% (Alveolata), and 30.1% (Rhizaria) (Fig. 2A). Correspondingly, they accounted for 6.6% (Stramenopiles), 71.9% (Alveolata), and 17.4% (Rhizaria) of the total ASVs. These three subclades displayed remarkable shifts in relative abundance across water depths (from the shallow to deep layers), indicating vertical variation in protist communities. Moreover, Chlorophyta were occasionally abundant in a few locations, mainly in the shallow and deep chlorophyll maximum (DCM) layers, with average contributions of 1.6% and 1% to the relative abundances and ASV numbers, respectively. The remaining groups were minor, with an average relative contribution of less than 1% in terms of the relative abundance or ASV number. In particular, Discoba, where the diplonemids fell, had an average percentage of 0.003%.

For the diplonemid assemblages derived from group-specific primers, 1,930,502 sequences and 24,783 ASVs were recovered (Table S1). Similar to the entire protist communities, for diplonemids, only a small fraction of the ASVs was shared by all four layers (Fig. S1). The four families, namely, Eupelagonemidae (formerly known as DSPD I), Hemistasiidae, DSPD II, and Diplonemidae, exhibited average relative abundances of 93%, 4.9%, 0.8%, and 0.7%, respectively (Fig. 2B). Meanwhile, a few unknown diplonemids accounted for 0.6% of the total population.

### Diversity estimation

**Fig 3:**
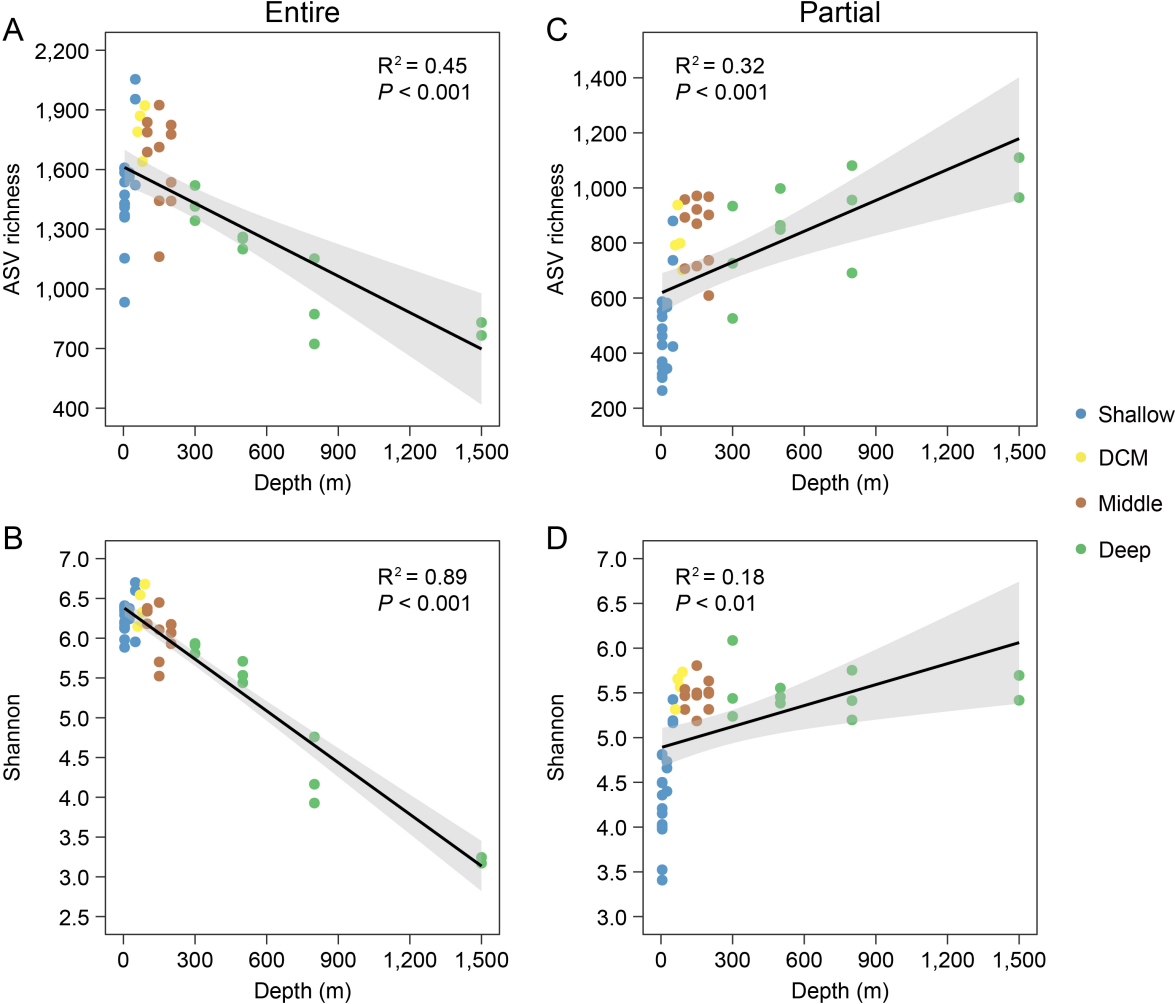
ASV richness and Shannon diversity of the entire (**A and B**) and partial (**C and D**) protist communities. The black lines indicate linearly significant relationships (*P* < 0.05), for which 95% confidence intervals (gray shadows) and R^2^ values are shown.

Both ASV richness and the Shannon index of the entire protist communities decreased significantly with increasing water depth (*P* < 0.05), whereas the opposite trends were observed for the diplonemid assemblages (Fig. 3).

**Fig 4:**
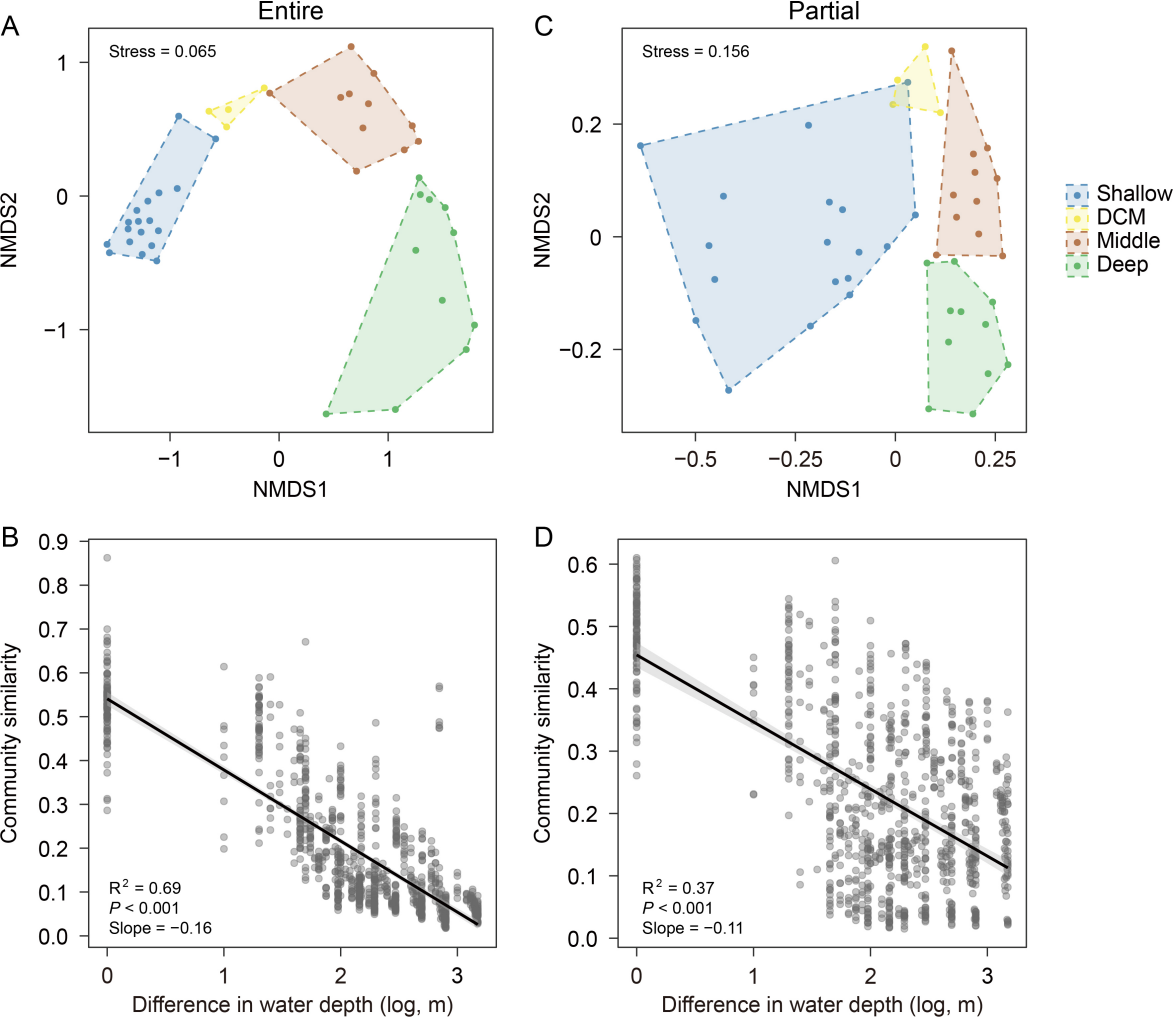
NMDS biplot and linear regression relationship of community similarity against the difference in (log-transformed) water depths of entire (**A and B**) and partial (**C and D**) protist communities. In panels A and C, the stress values are 0.065 and 0.156, respectively. In panels B and D, the black lines indicate linearly significant relationships (*P* < 0.001), for which the 95% confidence intervals (gray shadows), R^2^ values, and slopes are shown.

In terms of beta diversity, both the entire and partial protist communities showed significant differences among the four layers based on the permutational analysis of variance (PERMANOVA, *P* < 0.05) and Bray–Curtis dissimilarity; therefore, they were separated from each other in non-metric multidimensional scaling (NMDS) plots (Fig. 4A and C). The effect of water depth on shaping both the entire and partial protist communities was also confirmed based on the depth–decay relationship of community similarity (Fig. 4B and D), which indicated a decrease in community similarity with increasing water depth difference. Accordingly, canonical correspondence analysis (CCA) showed that a few major environmental variables contributed greatly to variations (*P* < 0.001) in both the entire and partial protist communities (Fig. S2).

Additionally, an unweighted pair group method with arithmetic mean (UPGMA) dendrogram for relatively abundant ASVs (relative abundance >0.5% and ranked in the top 30) showed that samples from different layers were generally clustered together (Fig. S3). This indicated that the vertically varying proportions of these ASVs largely contributed to the beta-diversity-related patterns observed across water depths.

### Phylogenetic structure of diplonemids

**Fig 5:**
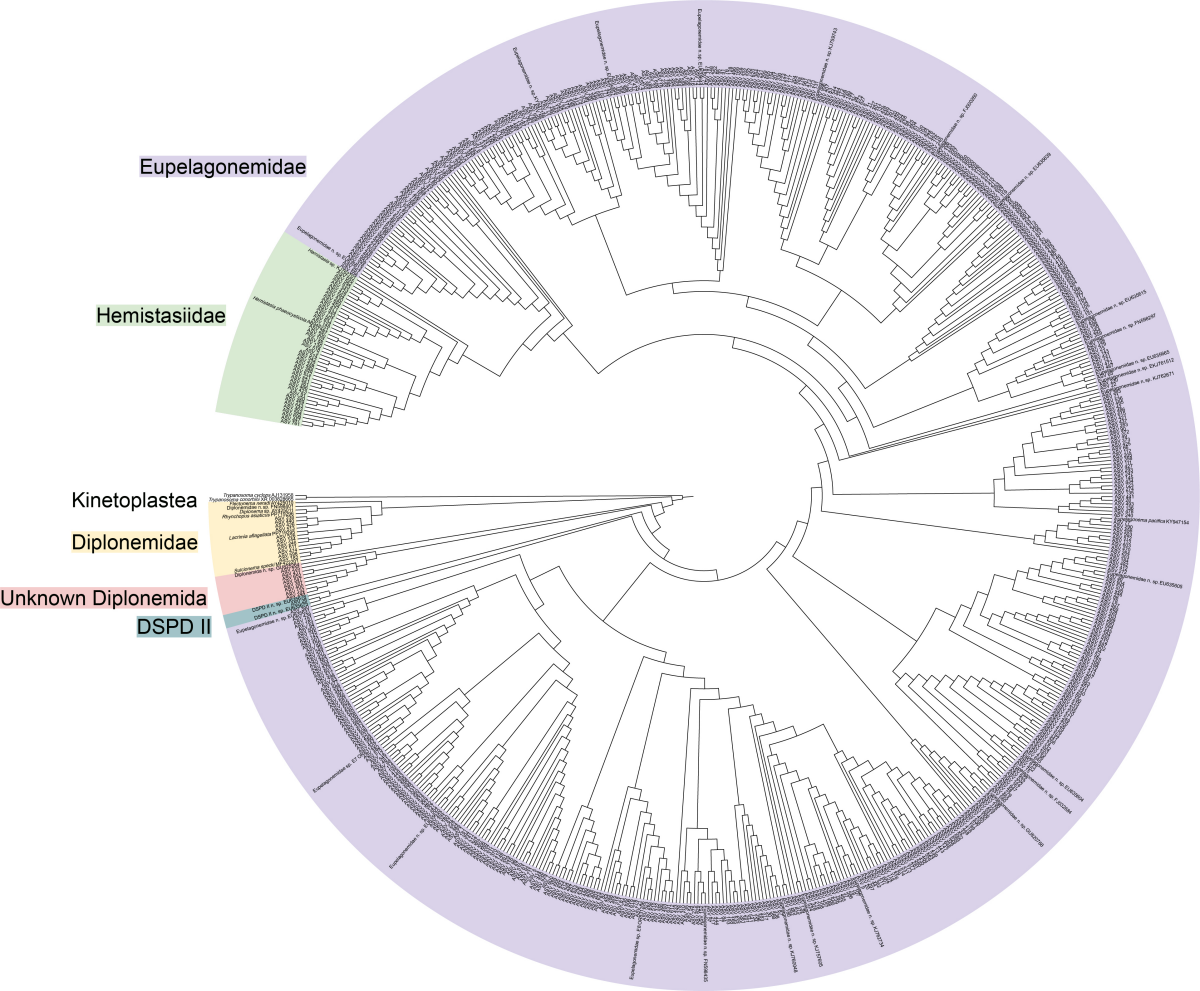
Maximum-likelihood tree of the diplonemid assemblage based on 650 ASVs and 36 references. Two Kinetoplastea sequences are used as outgroups. Many references are environmental sequences without species names and are thus defined as novel species (n. sp.) within a given family.

ASVs with an average relative abundance greater than 0.01% were used for phylogenetic analyses. A total of 650 ASVs were retained with an average proportion of 81.6%, suggesting that the selected ASVs generally accounted for the phylogenetic structure of diplonemids. Most phylogenies (*n* = 588) belonged to the family Eupelagonemidae (Fig. 5), while fewer representatives belonged to the other three families (Hemistasiidae, 42; Diplonemidae, 11; and DSPD II, 1). Notably, eight ASVs did not belong to any of the four families and were thus considered unknown Diplonemida.

### Quantitative estimation of diplonemids

**Fig 6:**
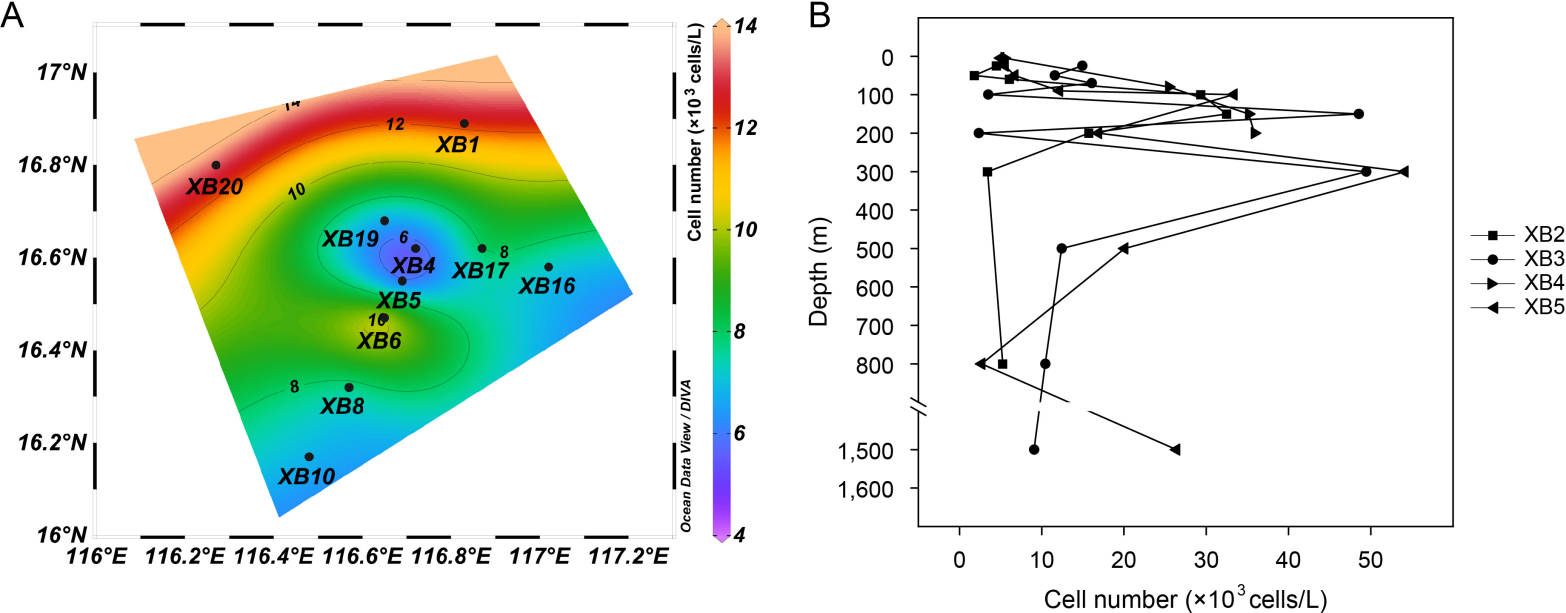
Horizontal (**A**) and station-based vertical (**B**) pattern of diplonemid cell numbers.

The 18S rRNA gene copy numbers of the diplonemids ranged from 3 × 10^4^ (Station XB2, 50 m) to 9.1 × 10^5^ copies/L (Station XB5, 300 m) (Fig. S4). Cell numbers based on copy numbers showed a horizontally broad range of 5 × 10^3^ (Station XB5, 5 m) to 1.3 × 10^4^ cells/L (Station XB20, 5 m) (Fig. 6A). Vertical variations were also remarkable, with the minimum (1.8 × 10^3^ cells/L) and maximum (5.4 × 10^4^ cells/L) values observed at depths of 50 m (Station XB2) and 300 m (Station XB5), respectively (Fig. 6B), accompanied by varying physicochemical conditions, as indicated by the temperature–salinity (T–S) diagram (Fig. S5). Moreover, compositional shifts were observed when comparing the cell number-based relative abundances with those of the sequencing reads (Fig. 2B; Fig. S6).

## DISCUSSION

In this study, we explored the diversity and compositional variations of both the entire and partial protist communities around the Xianbei Seamount, expanding the knowledge of the planktonic biome in this region, along with earlier studies (39, 40). We showed that the alpha and beta diversities of both the entire protist communities and diplonemid assemblages varied strongly across the vertical water columns in the surrounding waters. Diplonemid assemblages exhibited substantial diversity and complex phylogenetic characteristics. Furthermore, we found that diplonemids were abundant around the Xianbei Seamount, showing horizontal and vertical variability in cell numbers.

### Water depth-regulated community variation

Both the entire and partial protist communities showed water depth-regulated alpha diversity patterns (Fig. 3). Despite the strong environmental gradients related to water depth in pelagic oceans, significant linear regression relationships have rarely been observed for the alpha diversity of protists. This is partly because protists are involved in complex and vertically varied biogeochemical processes, such as carbon cycling (44). For example, protist communities typically comprise autotrophic (e.g., Chlorophyta), mixotrophic (e.g., Haptophyta), and heterotrophic lineages (e.g., Picozoa), as observed in our study (Fig. 2). Consequently, protist communities across water depths comprise numerous subclades with vastly different species diversity (45), leading to inconsistent vertical patterns (46–48). However, hydrographic conditions affected by seamounts may enhance the vertical connectivity of protist communities (19, 49). The locally specialized biogeochemical cycles of the Xianbei Seamount (40) may also have enhanced our linearly significant pattern of alpha diversity. Consistent with previous findings indicating that protists have higher diversity in epipelagic zones, our results further suggest that protists play significantly more diverse roles in biogeochemical processes in relatively shallow waters (down to a depth of 1,500 m) around the Xianbei Seamount.

Compared with the entire protist communities, the diplonemid assemblages exhibited the opposite trend in vertical alpha diversity. Initially, clone library-based studies found that diplonemids preferentially inhabited the deep oceans (24, 25); however, metabarcoding-based circumglobal surveys identified them throughout the water column, including the photic zone (29). Owing to these findings, the formerly defined deep-sea clade DSPD I was considered part of the subsequently erected family Eupelagonemidae (50). This was supported by our finding that the most abundant diplonemid representative (ASV 1) affiliated with Eupelagonemidae was widely detected in the shallow layer (Fig. S3B). Moreover, our findings regarding the trend of higher diversity in deeper waters are consistent with observations from other areas (34), confirming that diplonemids are relatively more diverse in deeper waters. Additionally, this diplonemid trend did not weaken that of the entire protist communities, considering that the universal primer set could not efficiently amplify the V4 region of the diplonemid 18S rRNA gene (51). The length (longer than 500 bp) of the diplonemid V4 region (29) hinders its recovery from a 2 × 250 bp paired-end sequencing platform (as used in this study) adapted to the universal primer set. Therefore, diplonemid assemblages are generally filtered out from the data of entire protist communities, which is the main reason for the simultaneous occurrence of these two contrasting alpha diversity patterns.

Water depth-regulated beta diversity patterns (Fig. 4A and C) have been observed in several studies (52, 53). Therefore, the major environmental factors accounting for the vertical gradients were explanatory in terms of community variation (Fig. S2). Moreover, as mentioned above, the entire protist communities include many clades with substantially different characteristics. For example, the two pigmented taxa ASV 21 and ASV 28, affiliated with *Ostreococcus* (Mamiellophyceae, Chlorophyta) and *Bathycoccus* (Mamiellophyceae, Chlorophyta), respectively, were disproportionately abundant in the DCM layer (Fig. S3A). Besides, both showed 100% sequence similarity to full-length clones previously detected in the photic zone across the South China Sea (54). Compared with the diplonemid assemblages, the entire protist communities varied more greatly across the water columns (i.e., they showed a broader range of beta diversity). Correspondingly, the entire protist communities had a steeper slope for the depth–decay relationship of community similarity (Fig. 4B and D), showing a strong pattern of marine microbial biogeography (48). The shared mechanisms driven by water depth led to a significant correlation between the beta diversities of these two categories (Mantel correlation coefficient *r* = 0.78), although the diplonemid assemblages were not well documented in the entire protist communities.

### Great diversity of diplonemids

The phylogenetic structure of the diplonemids recovered from the Xianbei Seamount (Fig. 2B and 5) was consistent with currently available knowledge. First, most phylogenies (90.8%) belonged to the family Eupelagonemidae (formerly known as DSPD I), which was assigned up to 97% of the total barcodes in the extended *Tara* Oceans data set (29). The prevalence of Eupelagonemidae in upper waters supports that this family is not restricted to deep-sea pelagic environments (31, 50). Notably, the family Hemistasiidae was mainly recovered from the shallow layer (28) and phylogenetically neighbors Eupelagonemidae (31, 42). With an average relative abundance of 11.3% in the shallow layers (Fig. 3B), our results differ from those of previous reports showing minor contributions from Hemistasiidae, despite its wide occurrence (34). In addition to the expected low relative abundance of DSPD II and Diplonemidae, an unknown clade was observed (Fig. 5), comprising certain ASVs closely related to the uncultured clone (accession number GU825264), which showed less than 90% similarity with others in GenBank (55).

In addition to those observed in the *Tara* Oceans expedition, diplonemids have been widely recovered from the Ross Sea (56), Ligurian Sea (33), and Weddell Sea (45) by targeting the V9 region. Whether the diversity of diplonemids was overestimated is debatable owing to the intragenomic variability of the V9 region (57, 58). Unfortunately, only a few studies have explored the diversity of diplonemids using the V4 hypervariable region (29), which is one of the two most widely employed regions for protist metabarcoding (13). Benefiting from the newly designed primers adapted to popular high-throughput sequencing platforms (42), our results support the great diversity of diplonemids from the V4 region. Compared with V9-derived sequences, which usually exhibit higher richness, especially at high taxonomic levels (59, 60), the V4 region is suitable for particular groups at low taxonomic levels (61, 62). More importantly, our group-specific primer-based data enabled the thorough examination of spatial variations in diplonemids (around the Xianbei Seamount) from the perspective of community ecology, taking interest in previous studies (35).

### Numerical quantification of diplonemids

In the sequencing era, the lack of quantitative estimations of cell numbers remains a major shortcoming in studies on microbial communities. For marine protists, attempts to quantify taxon-specific copy numbers for cell number-based quantification began 20 years ago (63). We found that diplonemids had a maximum cell number of approximately 5.4 × 10^4^ cells/L (Fig. 6), namely, five orders of magnitude lower than the cell density observed in laboratory cultures, which peaked at 6 × 10^9^ cells/L (64). However, their cell numbers were an order of magnitude higher than previous estimates obtained using fluorescent *in situ* hybridization in the North Atlantic Ocean (65). Despite potential overestimations in our eDNA-based counting, it cannot be ruled out that diplonemids may indeed be more abundant in seamount-affected waters. Horizontally, the cell numbers were low in the surface water close to the seamount summit, accompanied by current movements tracked by T–S diagrams (Fig. S4). Vertically, higher cell numbers were recorded at depths ranging from 150 m to 300 m, which is generally consistent with diplonemid dynamics based on the relative contributions to total protists (66). Moreover, it has been well recognized that varying rRNA gene copy numbers result in uncertainties in the profiling of protist communities (28), and the copy number-adjusted structures of diplonemid assemblages accordingly showed proportional shifts (Fig. S6). For example, the cell number-based relative abundance of Hemistasiidae in the shallow layer increased from a sequence number-derived proportion of 11.8% (excluding two samples owing to the unavailability of qPCR data) to 50.6% (Fig. 2B). Overall, our numerical group-specific quantification is useful for studying protists and further interpreting their ecological roles (such as prokaryotic removal in deep oceans) (67).

This study has a few limitations. First, the sample size was relatively small for examining the biogeographic patterns of both entire and partial protist communities. In particular, only four stations were sampled vertically, which led to insufficient information for tracking seamount effects. The sampling area was restricted to geographic locations generally around the seamount, which limited the comparative framework to differentiate protist communities in seamount and non-seamount regions. Moreover, our eDNA-based quantification had some uncertainties related to the number of diplonemid cells. This was because, on the one hand, the eDNA-based evaluation included dead cells or fragments. On the other hand, copy numbers are unavailable for most diplonemid species. Therefore, copy numbers per cell were roughly assigned when converting them to cell numbers of diplonemids. Regardless of these methodological limitations, this study sheds light on deep-sea protist ecology, with a special emphasis on seamount-associated ecosystems.

## MATERIALS AND METHODS

### Sample collection

Details of sample collection around the Xianbei Seamount are presented in our previous study (39). In summary, 44 water samples were collected from 12 sites between August and September 2021 and used for molecular analyses (Fig. 1). Four sites (XB2–XB5) were vertically sampled (at depths ranging from 5 m to 1,500 m), whereas only surface samples were taken from the remaining eight sites at a depth of 5 m. The samples were classified into four categories according to water depth: shallow (5–50 m), DCM (60–90 m), middle (100–200 m), and deep (300–1,500 m) layers, as defined in our previous study (39).

For each sample, 2 L of seawater was collected using a rosette conductivity-temperature-depth (CTD) system (Sea-Bird Scientific, USA) equipped with Niskin-type bottles (OceanTest Equipment, USA). Seawater samples were pre-filtered using a 200 µm mesh and then filtered through 0.2 µm polycarbonate membranes (Millipore, USA). Subsequently, the filters were transferred into tubes, frozen in liquid nitrogen, and stored at −80°C until DNA extraction. Moreover, environmental variables, such as fluorescence (for defining DCM), water temperature, salinity, dissolved oxygen, density, and nitrogen saturation, were measured during the expedition using appropriate probes fitted to the CTD system (40).

### Metabarcoding and sequencing

The details of DNA extraction are described previously (39). To characterize the protist communities, the V4 region of the 18S rRNA gene was amplified using the primer pair TAReuk454FWD1 (5′-CCAGCASCYGCGGTAATTCC-3′) and TAReukREV3 (5′-ACTTTCGTTCTTGATYRA-3′) (41). PCRs were conducted in a 50 µL reaction volume, comprising 1.25 U *Ex Taq* DNA polymerase (Takara Bio, China), 1× PCR buffer, 0.2 mM dNTP, 0.5 µM of each primer, and 1 ng templates. The thermocycling program was as follows: initial denaturation at 94°C for 5 min, 25 cycles at 94°C for 30 s, 47°C for 45 s, and 72°C for 60 s, and a final extension at 72°C for 5 min. The lengths of the PCR products were verified on a 1.5% agarose gel, and successful libraries were sequenced (2 × 250 bp paired-end) on a NovaSeq 6000 platform (Illumina, USA).

To separately examine the assemblages of diplonemids, their 18S rRNA genes were amplified using the primer pair of S616F_Cerco (5′-TTAAAAAGCTCGTAGTTG-3′) and S948R_Dip (5′-AATGAAGACATTCTTGTC-3′) (42) to obtain barcodes (approximately 510 bp, including the primer) located in the V4 region. The PCR mixture was similar to that used for the protist communities; however, more templates (approximately 10 ng) were added. The thermocycling program was as follows: initial denaturation at 95°C for 2 min, 30 cycles at 95°C for 30 s, 50°C for 45 s, and 72°C for 30 s, and a final extension at 72°C for 5 min. The PCR amplicons were sequenced on a NextSeq 2000 platform (Illumina, USA), and 2 × 300 bp paired-end reads were generated.

### Sequence data processing

Raw reads were trimmed using cutadapt (68) for primer removal and merged using the fastq_mergepairs function in the USEARCH software (69). The sequences were subsequently denoised into ASVs using DADA2 implemented in the QIIME2 pipeline (70) and singletons were removed. The taxonomic classification of ASVs was conducted using the PR^2^ database (version 5.0.0) (71). ASVs not affiliated with protists or diplonemids were discarded from the entire and partial protist community data sets, respectively.

### Diversity and community structure analysis

To visualize compositional variations across samples, the relative abundances at different taxonomic levels were determined. ASV tables were rarefied for alpha- and beta-diversity-related analyses. To estimate alpha diversity, the number of observed ASVs (i.e., richness) and Shannon index were calculated using the vegan package (72) in R (73). Venn diagrams were generated to show the overlapping and unique ASVs in the shallow, DCM, middle, and deep layers. Linear regressions were conducted to examine the relationship between alpha diversity estimates (ASV richness and Shannon index) and water depth, and the threshold for a significant relationship was set at *P* < 0.05.

To explore beta diversity, Bray–Curtis dissimilarity was calculated, and differences in the overall samples were visualized using NMDS biplots. PERMANOVA was conducted to assess the multilayer differences (999 permutations). CCA was performed to determine the effects of environmental factors with 999 Monte Carlo permutations. To explore community dissimilarities related to changes in water depth, the linear regression relationship of community similarity (1 − Bray–Curtis dissimilarity) against differences in the water depth was examined, and a significant estimate (*P* < 0.05) confirmed the depth–decay relationship of community similarity (48).

To illustrate the compositional variations across samples at the ASV level, we selected ASVs with an average relative abundance exceeding 0.5% and ranked in the top 30 (only 17 ASVs were retained in the entire protist communities). A UPGMA dendrogram was then generated based on the Bray–Curtis distances.

### Phylogenetic analysis of diplonemids

Phylogenetic analyses were conducted based on diplonemid ASVs with an average relative abundance higher than 0.01% (*n* = 650). Representative ASV sequences were aligned using MAFFT (74), and ambiguously aligned regions were trimmed using trimAl (75). Thereafter, a maximum-likelihood phylogenetic tree was constructed through the CIPRES portal (76) using RAxML-HPC2 on XSEDE with a GTR + Γ + I model and 1,000 bootstrap replicates, and the resulting tree was visualized using iTOL (77).

### Real-time qPCR of diplonemids

To estimate the (cell number-based) abundance of diplonemids, qPCR was conducted using a method modified from previous studies (63, 78). In summary, the primer set S616F_Cerco and S948R_Dip was used to amplify the rRNA gene fragments of diplonemids as described above. Subsequently, the PCR-generated fragments were purified using a MiniBEST Agarose Gel DNA Extraction Kit (Takara Bio, China) according to the manufacturer’s instructions, followed by cloning using a Clontech In-Fusion HD Cloning Kit (Takara Bio, China). The constructed plasmids were linearized using the *Hin*d III enzyme (Takara Bio, China). Thereafter, the linearized plasmids were run on an agarose gel, and their concentrations were measured using a Qubit Fluorometer (Life Technologies, USA). The number of rRNA gene copies was calculated as previously described (63). Five linear plasmids (based on 10-fold serial dilutions) were used to develop standard curves for the qPCR assays.

Fluorescent PCR was conducted on a CFX Opus 96 Real-Time PCR System (Bio-Rad, Singapore) using TB Green *Premix Ex Taq* master mix (Takara Bio, China). Each assay was conducted in triplicate and under the following thermocycling program: initial denaturation at 95°C for 30 s; 40 cycles at 95°C for 5 s, 55°C for 30 s, and 72°C for 30 s; and a final melting curve analysis. The R^2^ values of the standard curves were higher than 0.999, and the PCR efficiencies for the different assays ranged from 98% to 99%.

The resulting copy numbers were divided into different taxa according to their relative abundances by metabarcoding (79). Group-specific copy numbers were converted into cell numbers by combining previous estimates (80) and phylogenetic placements. In particular, for Diplonemidae and Hemistasiidae, estimates of 20.5 and 1.9 copies per cell were used, respectively, whereas an estimate of 17.2 copies per cell (median of presently estimated species) was applied for Eupelagonemidae, DSPD II, and unknown Diplonemida, for which species-based estimates were unavailable (80).

## Data Availability

Raw sequencing data are available from the National Center for Biotechnology Information (NCBI) Sequence Read Archive under PRJNA1313161.

## SUPPLEMENTAL MATERIAL

Supplemental material spectrum.02734-25-s0001.pdf Table S1 and Fig. S1 to S6.

ASM does not own the copyrights to Supplemental Material that may be linked to, or accessed through, an article. The authors have granted ASM a non-exclusive, world-wide license to publish the Supplemental Material files. Please contact the corresponding author directly for reuse.
