# Supplementary material for: Protist diversity and community structure around the Xianbei Seamount in the South China Sea
Source: Microbiol Spectr. 2025 Nov 24;14(1):e02734-25. doi: 10.1128/spectrum.02734-25 (PMC12772313; doi:10.1128/spectrum.02734-25)

Supplemental material

Table S1 Number of quality-checked reads and amplicon sequence variants (ASVs) in each entire and partial protist community.

Entire protist community			Partial protist community		
Sample	Reads	ASVs	Sample	Reads	ASVs
XB1	63,607	1,683	XB1	52,781	582
XB6	52,637	1,436	XB6	33,113	497
XB8	48,666	1,386	XB8	37,180	551
XB10	53,640	1,453	XB10	34,880	446
XB16	76,168	1,624	XB16	20,951	313
XB17	76,529	1,566	XB17	23,865	265
XB19	77,397	1,202	XB19	46,428	591
XB20	30,897	933	XB20	33,181	324
XB2.1	69,483	1,690	XB2.1	21,307	369
XB3.1	75,474	1,729	XB3.1	53,763	631
XB4.1	78,705	1,561	XB4.1	18,203	350
XB5.1	65,546	1,450	XB5.1	37,885	466
XB2.2	67,216	1,674	XB2.2	18,096	344
XB3.2	79,945	1,677	XB3.2	33,601	584
XB5.2	67,576	1,661	XB5.2	43,280	588
XB2.3	62,628	1,598	XB2.3	22,020	427
XB3.3	75,157	2,175	XB3.3	54,619	924
XB5.3	69,520	2,067	XB5.3	51,893	776
XB2.4	64,965	1,898	XB2.4	52,803	820
XB3.4	64,330	1,949	XB3.4	54,742	980
XB4.2	50,601	1,675	XB4.2	51,764	822
XB5.4	63,749	1,997	XB5.4	47,724	721
XB2.5	76,975	1,884	XB2.5	52,467	778
XB3.5	72,348	1,778	XB3.5	51,375	952
XB5.5	66,314	1,901	XB5.5	54,265	997
XB2.6	77,217	2,039	XB2.6	56,635	991
XB3.6	77,054	1,838	XB3.6	55,313	804
XB4.3	38,237	1,168	XB4.3	52,287	1,035
XB5.6	73,156	1,523	XB5.6	51,724	889
XB2.7	76,652	1,635	XB2.7	54,896	1,022
XB3.7	71,931	1,884	XB3.7	28,987	614
XB4.4	69,498	1,520	XB4.4	49,744	819
XB5.7	79,532	2,010	XB5.7	52,630	951
XB2.8	75,593	1,633	XB2.8	53,225	958
XB3.8	73,875	1,428	XB3.8	32,626	547
XB5.8	70,130	1,482	XB5.8	47,957	814
XB2.9	67,404	1,330	XB2.9	52,039	1,078
XB3.9	71,555	1,276	XB3.9	44,476	906
XB5.9	64,301	1,325	XB5.9	50,480	926
XB2.10	41,096	732	XB2.10	55,449	1,024
XB3.10	87,797	976	XB3.10	33,823	712
XB5.10	63,183	1,239	XB5.10	50,138	1,138
XB3.11	85,280	962	XB3.11	55,176	1,179
XB5.11	59,581	819	XB5.11	50,711	1,044

Fig. S1 Venn diagram showing the numbers of overlapping and unique amplicon sequence variants (ASVs) among shallow, deep chlorophyll maximum (DCM), middle, and deep layers in entire (A) and partial (B) protist communities.

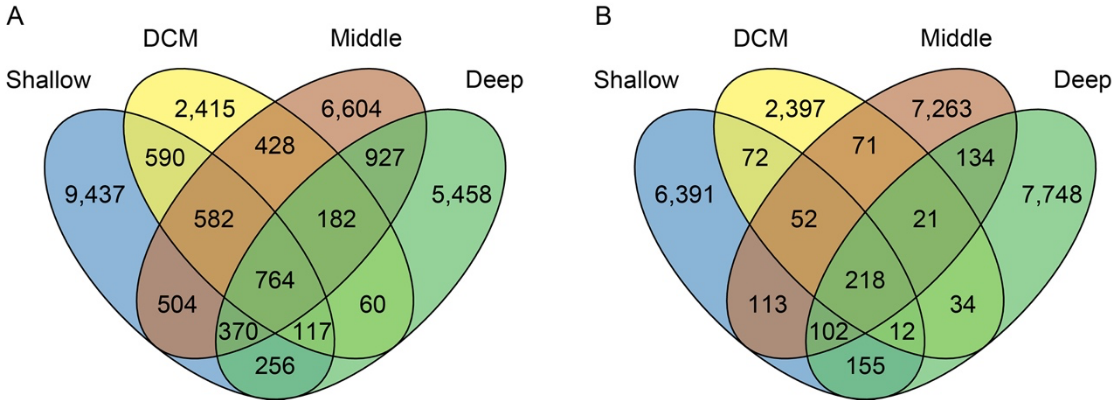

Fig. S2 Canonical correspondence analysis (CCA) biplot showing the correspondence of different environmental factors to the entire (A) and partial (B) protist communities. The significance ($P < 0.001$) of the explanatory power of each axis is based on 999 Monte Carlo permutations.

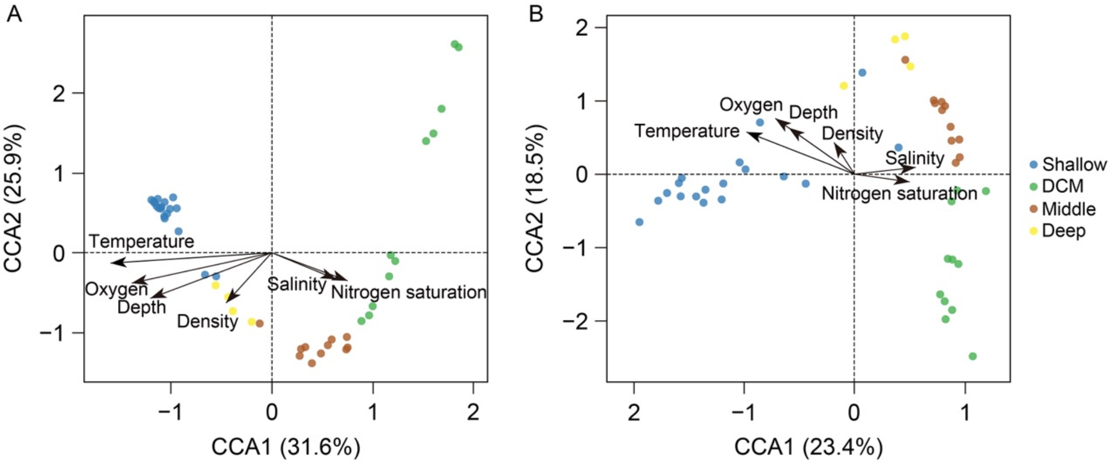

Fig. S3 Unweighted pair group method with arithmetic mean (UPGMA) dendrogram of subcommunities, comprising amplicon sequence variants (ASVs) showing an average relative abundance higher than 0.5% and ranked in the top 30. The relative abundances of each ASV in the entire (A, n =17) and partial (B, n =30) protist communities are shown in the barplots.

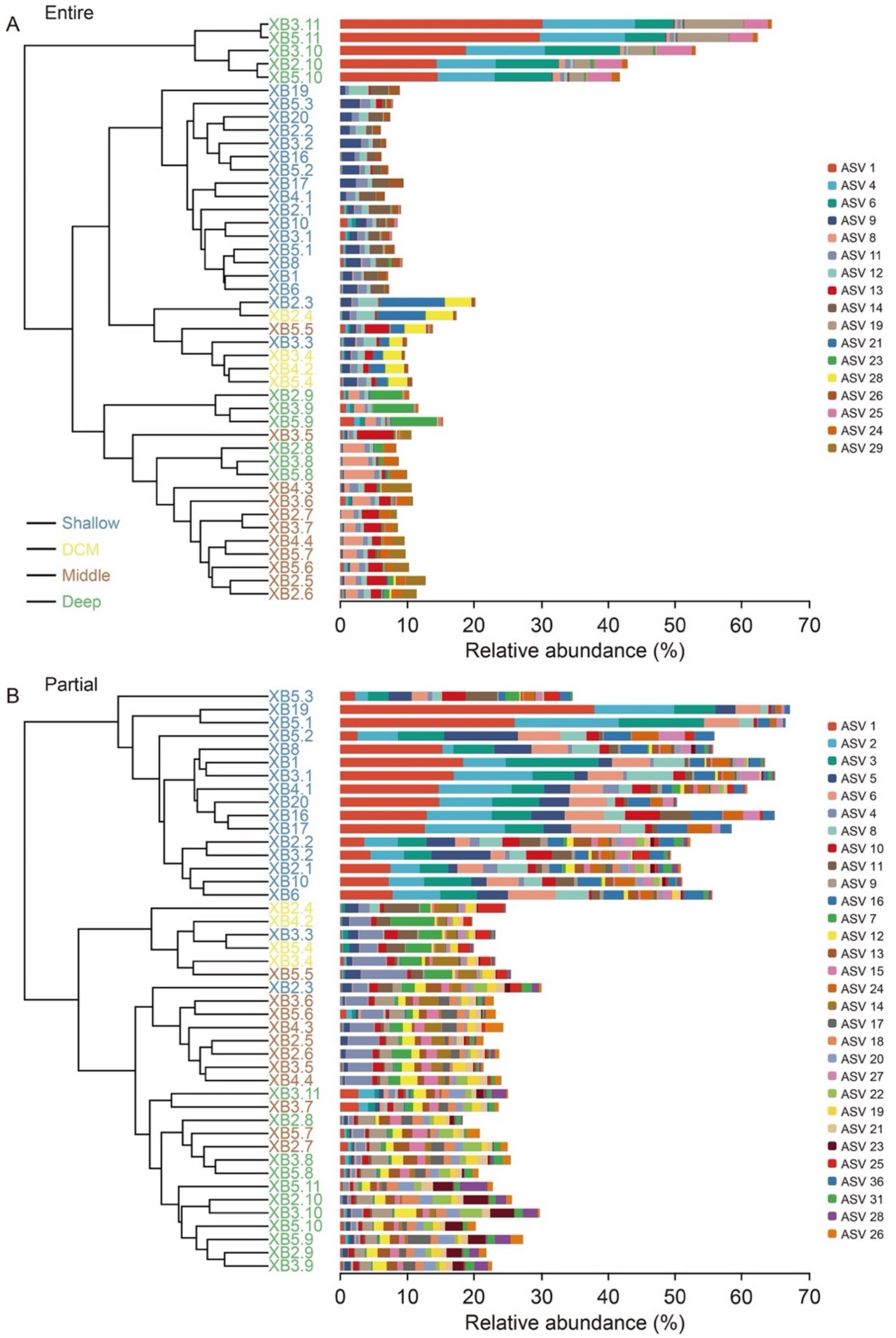

Fig. S4 Boxplot showing the 18S rRNA gene copy numbers of diplonemids in the four layers. Significant differences (*P* < 0.05) based on Tukey's honestly significant difference (HSD) tests are indicated by different letters.

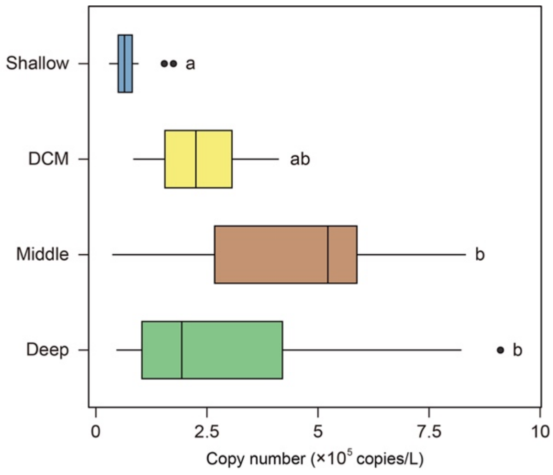

Fig. S5 Temperature–salinity (T–S) diagram of the four vertically sampled stations.

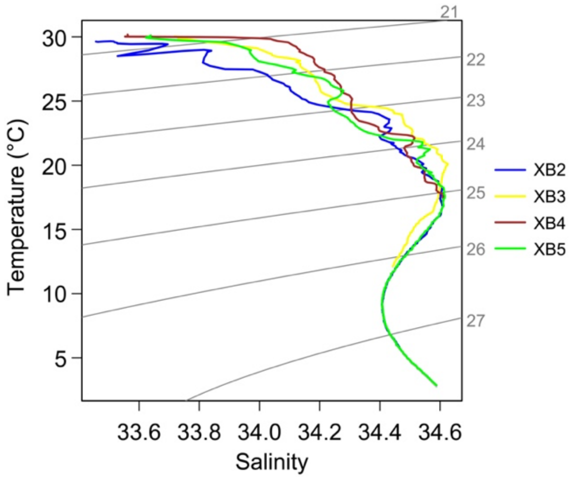

Fig. S6 Cell number-based compositions (in %) of diplonemid assemblages derived from 18S rRNA gene copy numbers. Blanks indicate an absence of data.

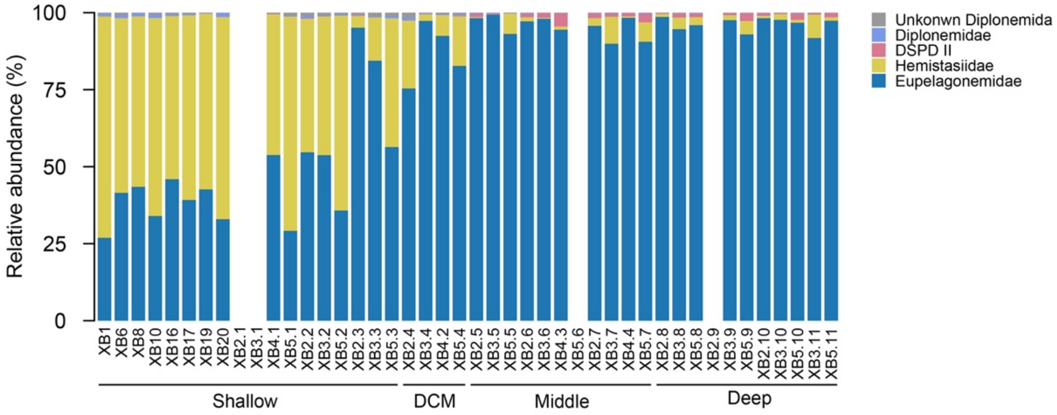

Supplement: Supplemental material — Table S1 and Fig. S1 to S6. [file spectrum.02734-25-s0001.pdf]
